# Unpleasant Results from the Use of Iodoform

**Published:** 1882-05

**Authors:** 


### Unpleasant Results from the Use of Iodoform.

Dr. Fifield wished to present to the attention of the Boston Society for Medical Improvement a case which had caused him much anxiety from the surprising effects produced by the cautious application of a small quantity of iodoform in powder to some ulcerated surfaces. In view of the great and constantly increasing use of iodoform, both in the hospitals of Europe and in this country, he deemed the subject worthy of much careful study, and thought that as the substance had been spoken of as a rival, and in some respects the superior, of carbolic acid as an antiseptic, its possible dangers as well as its good effects should be made known as quickly as may be. Dr. Fifield then alluded to the two fatal cases at Breslau, where iodoform had been largely and successfully used in the treatment of caries. The history of these cases showed death to have resulted in one case in two days, in a second in sixteen days. In both there was intermittent drowsiness ending in coma, paralysis of sphincters, aphonic disturbance of speech, contraction of the muscles of the neck, and scaphoid retraction of the abdominal walls, together with great frequency of pulse from the beginning. Temperature normal. In the second case the patient continued in good condition for nine days during the use of iodoform (externally), then headache, and somnolence for two days. Fatty degeneration of the heart with cloudiness of the liver and kidneys were shown post mortem.

Dr. Fifield's case was as follows:

A young lady had had for some time ecthymatous ulcerations with typical, piled-up, oyster-shell-like scabs in different parts of the body, particularly affecting the scalp, which was the seat of numerous foul ulcerations threatening the very bones beneath. On Friday last, at very urgent solicitations of relatives that some vigorous effort be made to arrest the progress of the disease which had obstinately refused to yield to mercurials and iodides in all forms, Dr. Fifield had removed certain scabs on the hands and one scab on the left temple, and had sprinkled them with iodoform. On Saturday, no unpleasant result having been experienced, the scabs of three other ulcers of the scalp had been removed and their surface lightly strewn with the same powder. On Saturday afternoon he was surprised to find the patient with swollen scalp, profuse serous discharge from nearly its whole surface, which presented a typical acute eczema. No constitutional effects complained of.

On Monday head and face enormously swollen, eyelids puffed out to a degree interfering with vision. Neck somewhat swollen on right side with tendency to contraction of muscles, profuse serous and purulent discharge from scalp, soaking the pillow on which the head rested. A powerful odor of iodoform filled the house. At noon the same day the eyelids were less swollen, the neck rather more.

Tuesday, 28th, swelling subsiding. Some little nausea complained of; had not rested well; pulse rather quick; temperature normal.

March 1st. All alarming symptoms have disappeared.

Dr. West, in answer to Dr. Fifield, said that Professor Billroth used iodoform very freely in the treatment of wounds, and out of hundreds of cases which he had seen treated in this way he had never seen a case of poisoning. The cases of poisoning were where cavities had been filled with iodoform.

Dr. H. J. Bigelow, referring to the use of iodoform in his wards, said that in certain subjects it acted as a local irritant, and in this way might cause trouble.

Dr. C. H. Williams said that he had treated an ulcerated eyelid with iodoform freely applied and without bad result.

Dr. Fitz spoke of the neçessity of preliminary cleanliness, as insisted upon by German authors, before applying the iodoform, —*Boston Medical and Surgical Journal.*

